# Controlling a Mouse Pointer with a Single-Channel EEG Sensor

**DOI:** 10.3390/s21165481

**Published:** 2021-08-14

**Authors:** Alberto J. Molina-Cantero, Juan A. Castro-García, Fernando Gómez-Bravo, Rafael López-Ahumada, Raúl Jiménez-Naharro, Santiago Berrazueta-Alvarado

**Affiliations:** 1 Departamento de Tecnología Electrónica, Universidad de Sevilla, 41011 Seville, Spain; jacastro@us.es (J.A.C.-G.); berrazuetasantiago@gmail.com (S.B.-A.); 2 Departamento de Ingeniería Electrónica Sistemas Informáticos y Automática, Universidad de Huelva, 21007 Huelva, Spain; fernando.gomez@diesia.uhu.es (F.G.-B.); ahumada@diesia.uhu.es (R.L.-A.); naharro@diesia.uhu.es (R.J.-N.)

**Keywords:** HCI, 2D cursor control, attention, blinks, Fitts’ model, emotion assessment

## Abstract

(1) Goals: The purpose of this study was to analyze the feasibility of using the information obtained from a one-channel electro-encephalography (EEG) signal to control a mouse pointer. We used a low-cost headset, with one dry sensor placed at the FP1 position, to steer a mouse pointer and make selections through a combination of the user’s attention level with the detection of voluntary blinks. There are two types of cursor movements: spinning and linear displacement. A sequence of blinks allows for switching between these movement types, while the attention level modulates the cursor’s speed. The influence of the attention level on performance was studied. Additionally, Fitts’ model and the evolution of the emotional states of participants, among other trajectory indicators, were analyzed. (2) Methods: Twenty participants distributed into two groups (Attention and No-Attention) performed three runs, on different days, in which 40 targets had to be reached and selected. Target positions and distances from the cursor’s initial position were chosen, providing eight different indices of difficulty (IDs). A self-assessment manikin (SAM) test and a final survey provided information about the system’s usability and the emotions of participants during the experiment. (3) Results: The performance was similar to some brain–computer interface (BCI) solutions found in the literature, with an averaged information transfer rate (ITR) of 7 bits/min. Concerning the cursor navigation, some trajectory indicators showed our proposed approach to be as good as common pointing devices, such as joysticks, trackballs, and so on. Only one of the 20 participants reported difficulty in managing the cursor and, according to the tests, most of them assessed the experience positively. Movement times and hit rates were significantly better for participants belonging to the attention group. (4) Conclusions: The proposed approach is a feasible low-cost solution to manage a mouse pointer.

## 1. Introduction

Several technical devices have been designed to assist people with communication-related disabilities. Such solutions must be specifically selected for each user, according to their motor skills. In general, they emulate the use of typical human–computer interaction (HCI) devices, such as keyboards or mouse pointers.

In comparison, a mechanical keyboard produces a higher information transfer rate than a mouse pointer; however, it needs a larger number of user commands. In its minimal configuration, a mouse only needs five control commands: to make the pointer move in four directions (upward, leftward, and so on) and to generate a left click for the selection. By controlling a mouse pointer, many computer applications can be managed—in particular, the virtual keyboards that most operating systems have in their accessibility options, which allow for typing. Some authors have proposed the control of mouse movements on a screen through ankle flexion [1] or with head movements. The authors in [2,3] used a webcam to track the head movement and several significant features in the face, such as the nose, whose position in the video was mapped into a cursor location on the screen. In this context, clicking can be accomplished by keeping the cursor still over the target for a certain duration (dwell time), or through the detection of some facial gestures, such as a smile, long blinks, winks, and so on. Alternatively, in [4], the authors proposed the use of a gyroscope placed on the head to control mouse movements, along with a consumer-grade electro-encephalography (EEG) headset for blink detection to emulate a mouse click.

Some people with severe disabilities are confined to a state in which it is not possible to accurately move their head or flex their ankle. For example, in advanced stages of diseases such as amyotrophic lateral sclerosis (ALS), people find it difficult to make the simplest movement, apart from controlling their eye movements [5] or blinking. Systems such as eye-tracker interfaces (ETIs) and brain–computer interfaces (BCIs) are suitable solutions for such people, as they do not need to have precise control over their eye muscles. Detecting blinking to generate a binary on/off output signal, in combination with an application that sequentially scans different ideograms/elements, may be enough for disabled people to access a computer. In fact, some authors have combined blinking detection with the scanning of different options, in order to move a mouse pointer on screen [6,7] and make a click, depending on winks or the duration of voluntary blinks.

### 1.1. Eye-Tracker Interface (ETI)

Many ETIs are based on the reflection of an infrared (IR) light on the surface of the eye. A camera tracks the pupil and the shiny IR spot in the eye. The relative position between them serves to determine the eye gaze—and then the cursor position—after an initial calibration process. The main drawback of this technology is the cost [8] and the so-called Midas touch effect [9], which consists of the random selection of an icon on the computer screen followed by the user’s gaze. Several low-cost and open-source solutions have emerged for eye tracking, with positioning accuracy similar to their proprietary counterparts [10]. For others, based on the use of only a webcam, the accuracy obtained is similar to that obtained by IR devices [11]. However, it is very difficult to set up such solutions and there is often no technical support.

### 1.2. Brain–Computer Interface (BCI)

There are several BCI modalities, depending on how the brain activity is measured, the type of information extracted, and the nature of the stimuli, including motor imagery (MI), steady state visual potential (SSVEP), and P300, among others. SSVEP requires several external visual stimuli, flickering at different frequencies. In [12], the flickering elements are shown around the cursor, making it easier to follow the cursor and not to lose focus. A variant is the so-called code-modulated visual evoked potential (c-VEP) [13], which uses a pseudo-random code to modulate the visual stimuli (not the frequency). The same authors have designed software to control any Windows application through a keyboard and mouse. To do so, they used 32 targets assigned with a 63-bit binary sequence with low auto-correlation, as each target used a circular-shifted sequence.

In another BCI modality, P300, the stimulus can be visual, auditory, or tactile, and is associated with an internal mental task that the subject has to perform (i.e., to mentally count) after an unlikely event occurs (i.e., a flash on an ideogram). Based on this paradigm, [14] proposed to control a mouse by displaying four randomly flashing squares on the screen, in order to represent the four directions of movement. Attention toward the flashes on one square by the user indicates the direction in which the cursor should move. Recently, it has been demonstrated that a P300 mouse emulation device can provide a promising alternative to a head mouse [15].

In motor imagery (MI), the BCI system is designed based on recognition of the EEG patterns generated when subjects imagine motion. The typical responses to such imaginary motion are event-related synchronization/desynchronization (ERS/ERD) [16]. A first approach to performing 1D cursor movements has been developed in [17,18]. People with or without motor disabilities can learn to control the amplitude of the alpha or beta rhythm and imagine limb movements [19] for positioning, or imagine keeping still for a while or clenching the hands in order to click [20], and so on.

Other BCI modalities include pattern recognition during mental tasks—for example, to compose several letters in the mind, the detection of which achieved an accuracy of 95% [21] and was applied to steer a wheelchair. In [22], the authors proposed the use of Dasher [23] with Emotiv. Subjects were trained to think of moving the cursor up, down, or being still. The exact algorithm used to convert such EEG signals into events is unknown, as the authors used proprietary functions offered by the Emotiv SDK.

The main drawback of BCI technology is the cost of the equipment used to capture the EEG signals, the training time for some BCI modalities, and the technical knowledge needed by the caregivers to set up the system.

### 1.3. Hybrid or Mixed Modalities

Studies have shown different ways to control a cursor on a screen, based on simultaneously including various BCI modalities. For example, in [24], the authors combined motor imagery and P300 to control vertical and horizontal movements, respectively. To support the P300 paradigm, the application used several flashing buttons placed at the computer borders, showing the direction of the movement. The same authors also studied the effect of substituting P300 with the SSVEP paradigm. For the selection action, the user must focus on a specific button and not think of moving their arms. Other authors have used mixed modalities to improve the performance of a single BCI. In [25,26], the SSVEP interface was improved by adding measurements related to the attention level or P300 potentials.

There also exist hybrid methods combining BCI with non-BCI technologies. Some studies have used EyeTrackers with BCI [27]—the former to control the cursor movements and the latter for selection, which allows for avoidance of the Midas effect. For example, in [28], subjects were trained to imagine wringing out a towel as a selection mechanism, while, in [29], they increased their concentration level when the cursor was over the target. A scanning procedure, identical to many communication boards, allows users to select the appropriate option. The authors of [30] have investigated the use of a gaze tracker with an SSVEP, combining probabilistic models for each input to improve the estimation of user intent.

### 1.4. Blinking as Part of the Cursor Control

Voluntary blinking detection generates an on/off signal that, along with a board that includes the scanning of its elements, thus showing the cursor’s movements and clicking, allows users to steer the pointer on the screen as a joystick [7]. These voluntary blinks can be distinguished from natural blinking, as they take longer, or when the user closes only one eye (wink).

Blinks are usually considered as artifacts when recording bioelectrical signals, such as EEG. However, some BCI systems have included them as part of the control algorithm. For example, the authors in [31] proposed a hybrid BCI system combining SSVEP, MI, and blinks to control a quadcopter. Blinking allows for switching between two different navigation modes, while SSVEP and MI allow for moving the quadcopter horizontally or vertically.

The authors of [32] presented a system that integrates a speller, a web browser, an e-mail client, and a file explorer using EEG and electro-oculography (EOG) signals. The mouse control method combines left-/right-hand motor imagery for horizontal movements of the mouse and blink detection for vertical motion and selection. The latter was accomplished through the scanning of several buttons, containing the actions to perform, placed around the screen.

An EOG-based brain switch has been used to activate/inactivate a P300 speller, when needed [33], by performing a triple blink. The user could then input sinograms by alternating P300 and double-blink tasks. This method demonstrated higher input rates than the traditional P300 speller.

### 1.5. Main Goals of This Study

In this paper, we propose the use of a single-electrode low-cost EEG headset, such as NeuroSky Mindwave (NM), to control a mouse pointer without the need for using any intermediate scanning application to make it move.

The position of the electrode, Fp1, allows for measurement of the electrical activity associated with both the eye and the left pre-frontal cortex, where some high executive functions—such as selective attention [34]—are located. The cursor is always in movement, and voluntary blinks are used to select between rotation and linear motion, while the level of attention of the subject regulates its speed.

As will be shown, this approach implies several novelties and advantages over other existing strategies. On one hand, the cursor control algorithm enables a novel, simple, and efficient combination of user attention and voluntary blinks using only one device (a single-channel EEG). On the other hand, this control strategy makes it possible for the user to adequately drive the movement of the cursor, with hardly any training. Finally, the implementation of this strategy in a low-cost system would allow for the use of the system in everyday situations, without expert supervision.

It is also worth noting that, in the end, the opinion of users is the key to accepting a specific technology. This is because other non-technical aspects, such as fatigue, price, frustration, training time, environment, and so on, are also taken into account [35]. For example, the authors of [36] demonstrated that a person with locked-in syndrome (LIS) was able to gain control over an EOG, an eye tracker, and an auditory BCI, but the user preferred to keep employing their low-tech communication method, using residual eye movements, due to their proficiency and that of their caregiver. For this reason, we not only investigate the abilities of users in steering the cursor, but also the emotions elicited by the use of this strategy as a measure of its possible acceptance.

Section 2 explains the elements used to build this system and the procedure utilized to control the cursor movement and make selections (mouse clicks). Finally, Section 3, Section 4 and Section 5 present the methodology, results, and discussion, respectively.

## 2. Materials

### 2.1. Hardware

**Figure 1:**
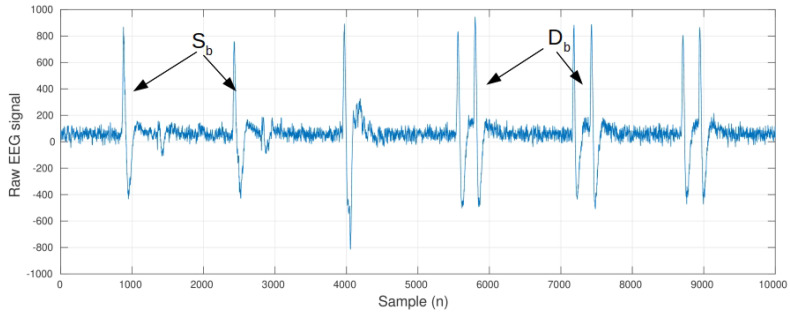
Raw EEG signal, with a sequence of artifacts associated with single ($S_{b}$) and double ($D_{b}$) blinks.

The NeuroSky Mindwave (NM) is a one-channel EEG headset that measures brain activity. This device features a unique dry steel alloy sensor (no medical gel needed) placed at the FP1 (according to the 10–20 placement system) position and a clip-type reference electrode placed at the left ear lobe (A1). NM delivers the raw signal at a sampling frequency of $F_{s}=512$ Hz (Figure 1). Besides the brain activity, the signal also contains several ocular artifacts, which stand out from the ’noisy’ baseline as high-amplitude waves, due to single or double blinks. These kinds of artifacts are very common in EEG recordings, as the eyelid serves as a ’sliding electrode’ that short-circuits the corneal positive charges to the frontopolar electrodes [37].

NM also delivers a proprietary index for attention at a rate of 1 Hz and in the range of [0,100]. The exact algorithm has not been published, although the manufacturer has stated that the index is strongly influenced by the β band. In fact, the attention level influences the EEG signal in several ways. First, it makes the signal more complex, such that its measurement could be accomplished by evaluating the fractal dimension [38]. Second, there also exist works that have studied the effect of attention on power bands [39], and the use of the ratio between θ/β—known as the Theta–Beta Ratio (TBR)—has also been reported as an indicator for attention deficit disorder (ADD) or hyperactivity disorder (ADHD) in patients [40]. Several studies have shown the feasibility of using NM for measuring the attention level [41]. In [42], it was shown that there exists a positive correlation between the reported attention level of this device and the self-reported attention levels in an assessment exercise. In [43], the single-channel EEG device accurately measured the overall level of mental attention in children with clinically determined developmental coordination disorders.

In comparison to other consumer-grade EEG headsets, such as Emotiv, NM provided worse results in terms of detecting cognitive loads, but it was preferred by users as it is more user-friendly, as well as easier to set up and maintain [44].

### 2.2. Cursor Control Algorithm

**Figure 2:**
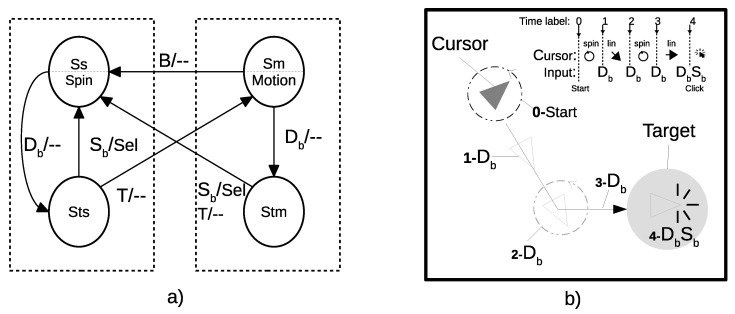
(**a**) FSM for cursor control. $S_{s}$ and $S_{m}$ describe the states where the cursor is spinning or moving straight on the computer screen. A double blink, $D_{b}$, makes the cursor stop temporally (states $S_{ts}$ and $S_{tm}$) and (1) generate a selection (Sel) if a third blink is detected in a period of time (T) or (2) continue the same type of movement. A border detection, *B*, stops the cursor movement. (**b**) Example of driving the cursor to a target, by means of a sequence of $D_{b}$ actions, and target selection by performing the sequence $D_{b}S_{b}$.

User EEG signals are captured by NM and transferred to a computer, where blinks are detected. Each blink detection is used as an input to an algorithm, implementing a four-state finite state machine (FSM) that controls the cursor movements on the computer screen (see Figure 2a). Two states, $S_{s}$ and $S_{m}$, represent the two possible cursor movements: rotation and linear motion, respectively. In general, isolated single blinks ($S_{b}$) are filtered out, as they may be due to the involuntary process of eye lubrication. Only double blinks ($D_{b}$) associated with a clear voluntary action by the subject, as well as single blinks produced immediately after a double blink, are considered as valid inputs. A $D_{b}$ input allows for changing between the two cursor movements. Nevertheless, this change is not immediate. Two other states, $S_{ts}$ and $S_{tm}$, are included in between, in order to temporally stop the cursor for a period of time, *T* (900 ms), and allow users to perform accurate target selection. If no blinks are detected during this time, FSM evolves either from $S_{ts}$ to $S_{m}$ or from $S_{tm}$ to $S_{s}$. However, if a new single blink $S_{b}$ is received before time *T* is over, FSM considers that a selection has been delivered and it evolves to the $S_{s}$ state (a user selection is also called a click, due to the similarity of this action to clicking a mouse pointer). In other words, a $D_{b}$ input makes the cursor change from rotating to linear motion and vice versa. Furthermore, for a click, it is necessary to detect a sequence of at least three consecutive blinks. Figure 2b illustrates an example of cursor driving and target selection, using the sequence $D_{b}/D_{b}/D_{b}/D_{b}S_{b}$. We did not consider blocking the effect of blink bursts in the FSM, as more than five fast blinks, which will cause unexpected behavior of the cursor under the user’s perspective, seems to be highly unlikely. $S_{s}$ is the initial state, but it is also a destination state if a user selection is delivered or the cursor arrives at the scenario’s border while moving forward (*B* input in Figure 2a).

We applied the algorithm published in [45] for the detection of single blinks. It is based on obtaining two main features from the raw EEG signal: (1) the difference between the maximum and minimum value in the epoch and (2) the energy of the ’blink-free’ EEG signal resulting from removing the baseline obtained by applying a Savitzky–Golay low-pass filter (order 2 and length 35) to the EEG epoch. With these two features, the algorithm is able to identify neural activity, blinks, or motion artifacts. The accuracy in detecting a blink is 98%. For the sake of simplicity, the detection of double blinks ($D_{b}$) was not included in the FSM shown in Figure 2. In fact, there are two other states between the trajectories that join the spin and motion states ($S_{s}$ and $S_{m}$) with their respective waiting states ($S_{ts}$ and $S_{tm}$). Double blink detection is delivered when two single blinks are detected in a period of time of 900 ms.

Moreover, the linear velocity, *v*, of the cursor on the screen can be modulated by the attention level, according to Equation (1), where AL is the normalized attention level, AL∈[0,1]; f(·) is a piecewise real-valued function defined by Equation (2), which linearly maps a specific range of AL, [0.3,0.7], into the cursor velocity; and $v_{max}$ and $v_{min}$ are the maximum and minimum cursor velocity, respectively. The variable $v_{min}$ was arbitrarily set at half of the maximum velocity, $v_{max}$:

(1) $$\begin{array}{c} v=v_{min}+f\left(AL\right)\left(v_{max}-v_{min}\right), \end{array}$$

(2) $$\begin{array}{c} f\left(AL\right)=\left\{\begin{array}{cc} 0 & if\;AL\leq 0.3\\ \frac{AL-0.3}{0.4} & if\;0.3<AL\leq 0.7\\ 1 & if\;AL>0.7 \end{array}\right.. \end{array}$$

The maximum velocity must be linked to the time that the user needs to perform a double blink. A high value in this variable has the effect of reducing the available time for the user to stop the cursor on the target (in linear motion) or at the appropriate angle (when it is spinning). Let $t_{D}$ be the time taken to perform a double blink. Assume that the target is a circle of radius *R*, given in pixels, as well. According to Equation (3), at maximum linear speed, $v_{max}$, the cursor could be stopped inside the target in $t_{D}$ seconds from any entry point, whereas the trajectory has an incident angle in the range of ±60°, which represents 66.7% of all possible incoming trajectories. On the other hand, cursor rotation consists of a sequence of discrete turns of $\theta_{step}$ degrees. Consequently, the user must have enough time to make a double blink between cursor movements. This limits the angular frequency to the value given by Equation (4):

(3) $$\begin{array}{c} v_{max}=\frac{R}{t_{D}}, \end{array}$$

(4) $$\begin{array}{c} \omega =\frac{\theta_{step}}{t_{D}}. \end{array}$$

To estimate the value to assign to $t_{D}$, we used the data given in [46]. They conducted several experiments to analyze blink detection with different devices, including the same EEG headset as ours. They found that, on average, a double blink takes 456 ms, with a standard deviation, σ, of 131 ms. This means that an assignment of the average value plus 3σ in $t_{D}$ would represent 99% of all double blink durations in the study. For this reason, we set $t_{D}$ to 900 ms.

**Figure 3:**
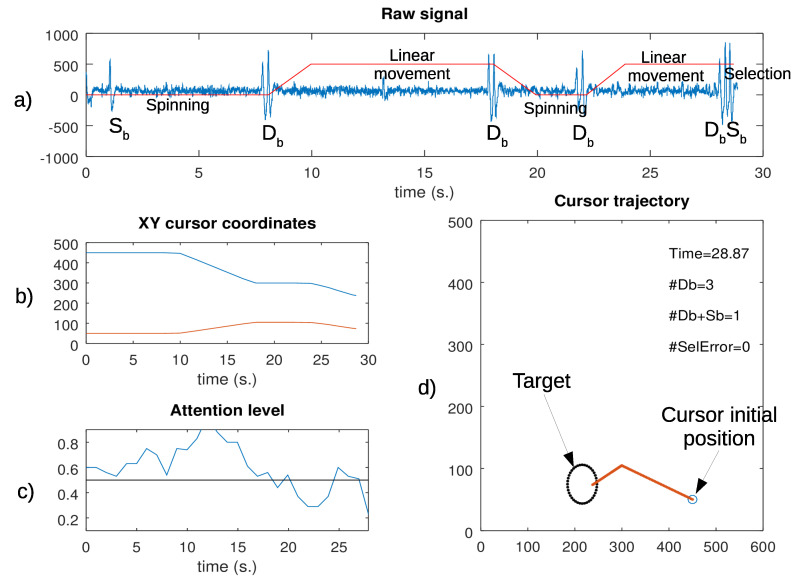
Signals captured during a trial: (**a**) raw signal, with a description of the type of cursor movement; (**b**) cursor coordinates; (**c**) attention level signal; and (**d**) cursor trajectory on the screen, showing its initial position, the target, and the end position.

Figure 3 depicts the signals captured during the experiment. Note that the double blinks allowed the user to switch between spinning and linear displacement. A final triple blink led to the target selection. The attention level index and the cursor trajectory are also shown.

## 3. Methods

### 3.1. Participants

Twenty (20) people (6 women, 14 men) were recruited for the experiment. Their ages ranged from 16.50 to 67.81 years (38.73 ± 15.40) at the time of the experiment, and 85% of them were right-handed, which is in concordance with the left-handed population [47].

All participants were previously informed about the details of the experiment and signed a consent form before taking part in it. The ethics committee of the regional government in Andalucía (Spain) approved the experiment.

### 3.2. Experiments

Participants were randomly distributed into two groups: $G_{na}$ and $G_{a}$. The group $G_{na}$ performed the experiment using only blinks to steer the cursor, so it always moved at the same velocity, regardless of the attention level (AL was set to 0.5 in Equation (2)). For the second group, $G_{a}$, the cursor speed was modulated according to the attention level. The average age in $G_{a}$ was 39.52 ± 14.76, whereas in the $G_{na}$ group, it was 37.55 ± 17.29. The difference between mean ages in the two groups was not significant (*p*-value = 0.8166, Wilcoxon’s test).

**Figure 4:**
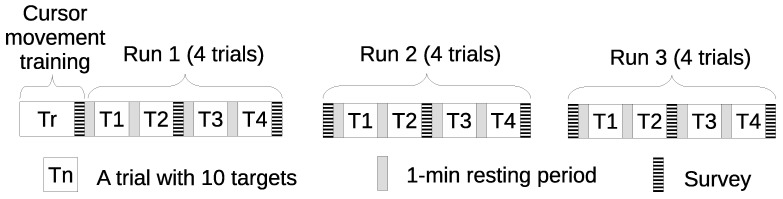
Experiment timing. All participants attended three different days to perform four trials consisting of selecting 10 targets, which were randomly screened. Between trials, there was a brief resting period of one minute.

The experiment comprised three runs, distributed on different days (see Figure 4). First, the experimenter showed the participants a short video containing explanations about how to carry out the cursor control and modulate the attention level. After watching the video, the experimenter also answered any questions from participants, when appropriate. Then, the participants, regardless of the group they belonged to, trained for 10 min. Here, the goal was that participants were familiarized with the control mechanism to move the cursor, make selections through blinks, and, for the $G_{a}$ members, learn how to modulate their attention level to speed up the cursor’s linear movements. Next, in each block, they performed four trials, consisting of moving the cursor towards ten different targets (and their selection), as soon as possible. Target sizes and distances to the cursor were randomized, in order to avoid any learning effect on the index of difficulty (ID), as explained below in Section 3.3. Between trials, there was a short resting period of 1 min.

**Figure 5:**
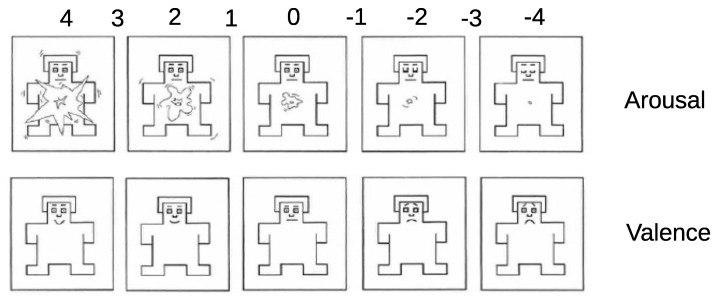
The SAM test showed every two trials to gain knowledge about the evolution of the internal state of participants during the experiment.

We used self-assessment manikin (SAM) tests to identify the emotional state of participants during the experiment. Figure 5 shows the tests used for assessing the arousal and valence dimensions of Russell’s model [48], coded by a 9-level Likert scale. In such a model, emotions can be represented as points in a two-axis coordinate system. As an example, happiness belonged to the first quadrant (with valence and arousal positives), while sadness was placed opposite to happiness (with valence and arousal both negative). To determine how the feelings of participants evolved during the experiment, the SAM test was used at the beginning of each run and every two trials. The watched video also contained guidelines about how to fill in the different surveys. Moreover, participants were asked to add any comments or adjectives (e.g., frustration, boredom, tiredness, and so on) at the end of each block, in order to obtain a more complete description of their feelings during the use of the proposed cursor control method.

On the last day, participants were given a questionnaire with three 5-level Likert-scale questions, ranging from 1 (very low) up to 5 (very high); see Table 1.

Participants were placed in front of a computer screen (23 inches) in an armchair (at a distance between 50–60 cm) and the lighting and room temperature were kept at comfortable levels. During the experiment, nobody could come into the room, in order to avoid any source of distraction, and an experimenter was always near the participant, in the same room, to supervise and ensure the correct execution of the trial. A webcam placed on the top of the computer screen recorded the whole session.

**Figure 6:**
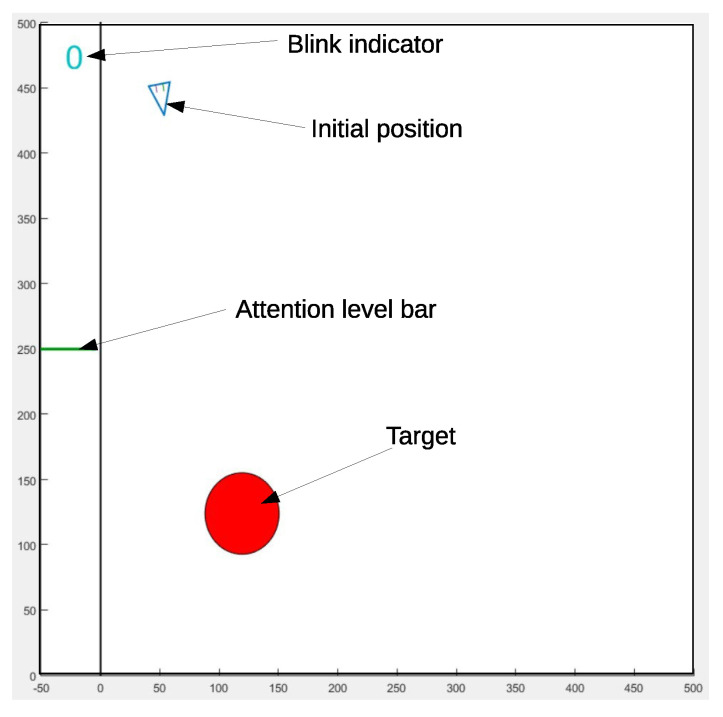
Matlab GUI interface, showing the working area with the red target and the cursor. On the left side of the screen, a line moves up and down according to the attention level. On the top, a blink indicator shows the number of blinks detected in the last period of time.

A Matlab GUI (Figure 6) was built to implement the cursor and targets on the screen. The targets were shown as red circles, with two variable sizes ranging from one-sixteenth up to one-eighth of the working area side. Their positions randomly changed when the cursor selected them. Once the target was placed at a new position, the cursor appeared away from it, at a distance ranging from one-sixteenth up to one half of the working area side, and with a relative angle that was a multiple of π/6 (see Table 2). Users could reach any target by performing the same minimal sequence of blinks: $D_{b}/D_{b}S_{b}$. If a selection was made outside of the target, a beep was sounded, as feedback. If the time taken in reaching the target was higher than 100 s, the software repeated it, such that the collection of trials with similar temporal constraints was guaranteed. The $v_{max}$ was dependent on the target’s size and, for an HD resolution monitor (1920 × 1080), this velocity was 128 px/s and 64 px/s for the large and small targets, respectively. The angular frequency, ω, was approximately set to 0.25 rad/s, which means that the cursor took around 25 s to complete a turn. The user interface also contained a lateral panel, with feedback indicators for blink and attention level.

An additional Java application registered, recorded, and synchronized all the information generated during the experiment [49]: the NM raw data, cursor coordinates, FSM state, blinks, and attention level. Hence, the whole experiment can be reproduced offline for further analysis. This application is based on labstreaming layer (LSL) [50], a multi-platform and multi-language library that allows for the synchronization of applications and devices through data streams. Video frames were also synchronized to LSL, allowing the researcher to see the scene associated with data segments, when this action was necessary.

### 3.3. Metrics

Fitts’ law [51] is a predictive model of human movement, used in human–computer interaction and ergonomics. It is a useful tool for modeling the movement time (MT), the time required to reach a target when performing tasks such as pointing with a mouse on a computer screen. Shorter values of MT are usually desirable for a given task; however, this fact depends on the difficulty of the task. Traditionally, MT is related to the index of difficulty (*ID*), given in bits, which, in turn, depends on the distance to the target, *D*, and its size, *W* (Equation (5)).

(5) $$ID=log_{2}\left(\frac{2D}{W}\right).$$

MacKenzie [52] proposed another alternative for the definition of *ID*, based on Shannon theory. This new formulation (Equation (6)) solves some of problems related to the original formulation, as it does not return negative results when using low *ID* values.

(6) $$ID=log_{2}\left(\frac{D}{W}+1\right).$$

**Figure 7:**
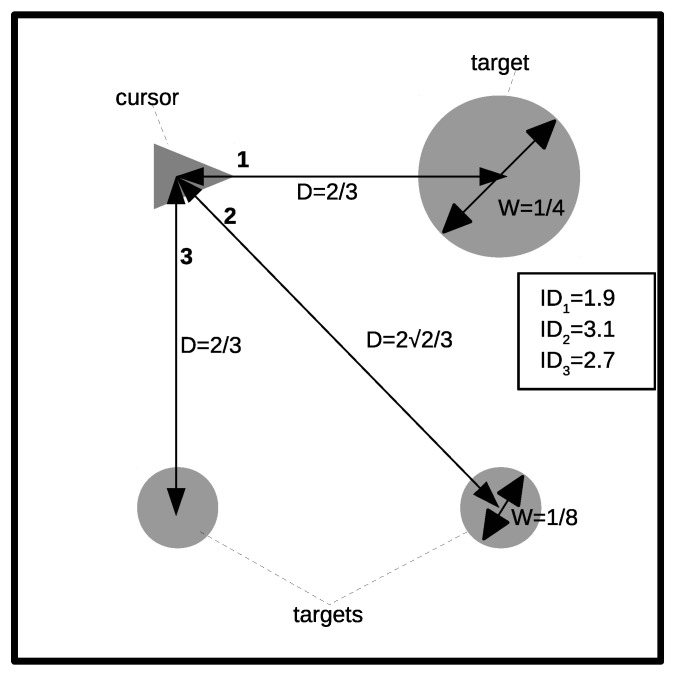
Illustration of the procedure followed to obtain a Fitts’ model. Targets with two different diameters were placed at several distances from the cursor. Fitts’ model establishes a linear relationship between movement time and ID. From the model, the IP—a metric for comparing computer pointing devices—can be obtained. See the text for more details.

In each experimental session, the radius, R, or width of the target (*W* = 2R), and the distance between the center of the circle and the initial position of the cursor, *D*, changed several times. Table 2 summarizes the main features of the experiment, including *ID*, which ranged from 1.22 up to 3.01 bits, which was within the range of many HCI systems. Figure 7 illustrates some experimental cases, with three targets placed at several distances from the cursor. The *ID* for each case is also shown. The two *ID*s in Table 2 were repeated in the experiment (marked with an asterisk in the table). This was carried out because the cursor speed was dependent on the target width, and different movement times were expected for the same *ID*. Distances were selected by considering that the targets would be placed on the center of a cell in a 3 × 3 grid covering the whole screen window.

The movement time and index of difficulty followed a linear relationship that can be characterized by Equation (7), where the parameters *a* and *b* can be empirically obtained by linear regression between the values of movement time (*MT*) and *ID* measured during the experiments. The value of *b*, or its inverse (which is called the index of performance, IP=1/b), can be used as a metric when comparing computer pointing devices.

(7) MT=a+b×ID.

Fitts’ law was originally employed to model one-dimensional movements; however, later on, it was also extended to two dimensions. If the targets are circles, then the effectiveness of the 1D model remains in 2D scenarios. For other target shapes, several adaptations and extensions of Fitts’ law have been developed. For example, in [53], it was shown that, when using rectangular targets, the best correlation between the model and experimental data was obtained when the parameter *W* in Equation (6) is the smaller side of the rectangle. ISO 9241-411 [54] describes the main performance measure for non-keyboard input devices: throughput (T, in bits/second or bps), which is calculated over a sequence of *n* trials as the ratio between the effective index of difficulty ($ID_{e}$), and MT, averaged for all difficulty levels (Equation (8)).

(8) $$T=\frac{ID_{e}}{MT}=\frac{1}{n}\sum_{n}\frac{ID_{e_{n}}}{MT_{n}}.$$

In contrast to ID, $ID_{e}$ takes into account the average performance of users. The calculation of $ID_{e}$ includes the effective distance, $D_{e}$, and the effective target width, $W_{e}$ (Equation (9)). $D_{e}$ is related to the real distance used by users until a correct target selection is achieved. It is obtained by computing the average of the length of the cursor’s paths, considering all the experiments. $W_{e}$ reflects the accuracy of the users in the target selection. It is computed under the assumption that the cursor’s position in the target selection follows a normal distribution. The Welford correction [55] is applied to adjust the target width to the experimental data, by assuming that the error rate is kept under 4%. This means that $W_{e}=4.133\sigma$, where σ is the standard deviation of the experimental data and 4.133 is a constant derived from a normal distribution, which guarantees that 96% of such data represent the real target width [56]. To this end, as targets with the same ID can be shown at different positions on the screen, the first step is to move them, together with the click coordinates, to the same screen position. Then, the average of click coordinates is used as the center of the effective target, whose radius is selected by gathering 96% of the click positions.

(9) $$ID_{e}=log_{2}\left(\frac{D_{e}}{W_{e}}+1\right).$$

Based on the recorded information, some additional metrics were obtained: the normalized effective distance (NED), which relates the optimal path (mp) to the real trajectory (rt), as shown in Equation (10) (the NED is a good indicator for comparing the motions performed by the user to the optimal route); movement variability (MV) [57] represents the extent to which the sample points lie in a straight line along an axis parallel to the optimal path (Equation (11) shows the expression, assuming that the optimal route is y=0 and *n* is the number of samples in the real trajectory); movement error (ME) is the average deviation of the sample points from the optimum path, regardless of whether the points are above or below (Equation (12), assuming y=0 as an optimal path); movement offset (MO) is the mean deviation of sample points from the optimum path (Equation (13), assuming y=0 as the optimal path), which indicates the tendency of the pointer to move “left” or “right” from the optimal path; the number of extra actions (EA), which represents the average number of extra actions needed to achieve the goal; and the hit rate (HR), representing the accuracy or percentage of achieved goals (true positives, TP), with respect to attempted targets (true and false positives, TP + FP).

(10) $$\begin{array}{c} NED=\frac{rt}{mp}, \end{array}$$

(11) $$\begin{array}{c} MV=\sqrt{\frac{\sum {\left(y_{i}-\bar{y}\right)}^{2}}{n-1}}, \end{array}$$

(12) $$\begin{array}{c} ME=\frac{\sum |y_{i}|}{n}, \end{array}$$

(13) $$\begin{array}{c} MO=\frac{\sum y_{i}}{n}. \end{array}$$

## 4. Results

### 4.1. Learning Effect on Main Indicators

**Figure 8:**
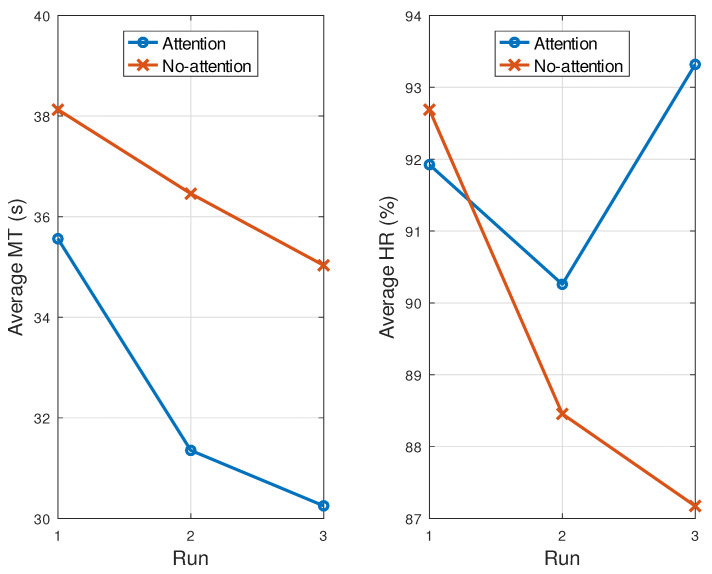
$\bar{MT}$ and $\bar{HR}$ evolution through different runs. Data were collected from Table 3.

It was expected that participants would improve their skills in controlling the cursor, showing lower MT and higher HR values at the end of the experiment. Table 3 presents these values for the $G_{a}$ and $G_{na}$ groups, averaged by trial and run, while Figure 8 shows the MT and HR grand averages, in order to visualize their tendency over time and, hence, any possible learning effect.

As can be seen, $\bar{MT}$ was lower in the last run than in the previous ones for both groups. This trend was significant in $G_{na}$, whose slope was statistically different from zero, according to the regression test. This proves the existence of a learning effect (at least, in this group).

The other variable, $\bar{HR}$, showed an unexpected trend in $G_{na}$. Instead of having a positive slope, the trend was negative and, so, $\bar{HR}$ scored the lowest in the last run. Nevertheless, by analyzing the confidence interval for the slope of the regression line, this tendency was not significant, which allowed us to conclude that there was no learning effect, or that it was not significant. For $G_{a}$, the final $\bar{HR}$ was the highest in the last run—which was expected—but the trend over time was irregular, decreasing from run 1 to run 2, then increasing from run 2 to run 3. There was no significant tendency either.

We also observed that $\bar{MT}$ increased in each run as the trials progressed (Table 3). Thus, $\bar{MT}$ was higher at the end of every run (i.e., in trial T4) than at the beginning (i.e., T1). This trend was statistically significant for the $G_{a}$ and $G_{na}$ groups in two out of the three runs (1 and 3). With regard to $\bar{HR}$, the last trial obtained a slightly lower rate than the first run, although there was no clear tendency over trials, as confirmed by the statistical analysis. Only for $G_{na}$, in the last run, was there a significant negative trend.

Hereafter, only the data associated with run 3 (i.e., the last run) are processed. This is the usual procedure in studies involving HCI models, in which the initial learning effect is removed from the results and final analysis. Different subscripts are added to $\bar{MT}$ and $\bar{HR}$, in order to distinguish the data set used. In this way, the subscripts ‘*a*’ and ‘na’ identify the averages obtained from all trials for the groups $G_{a}$ and $G_{na}$, respectively. Sometimes, the ‘*f*’ subscript is used to indicate that the averages were obtained with data from trial T1 (the fastest), in order to show the best performances that the proposed approach can hypothetically achieve. As an illustration, ${\bar{MT}}_{fa}$ represents the average of MT for T1 in run 3 for $G_{a}$, while ${\bar{HR}}_{na}$ is the mean for all trials in run 3 for $G_{na}$.

### 4.2. Index of Performance (IP) and Throughput (T)

Fitts’ model establishes a linear relationship between $\bar{MT}$ and the ID, in which the index of performance (IP) represents the inverse of its slope. We obtained four different models overall, covering the two groups (Attention and No-Attention) and two conditions (fast and averaged). Table 4 shows the MT for $G_{a}$ ($MT_{a}$, $MT_{fa}$) on the averaged and fast conditions, indexed by ID and their counterpart results ($MT_{na}$, $MT_{fna}$) for $G_{na}$.

**Figure 9:**
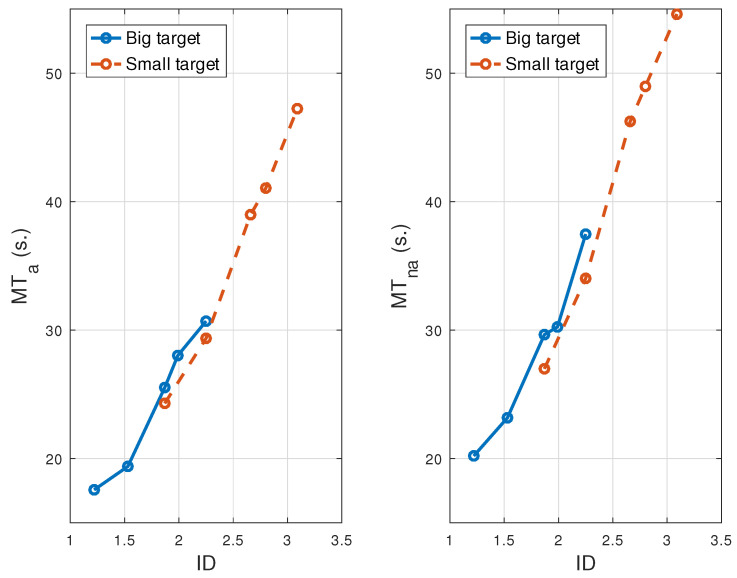
Dependence of ${\bar{MT}}_{a}$ and ${\bar{MT}}_{na}$ on ID and target size for all trials.

Figure 9 shows plots for ${\bar{MT}}_{a}$ and ${\bar{MT}}_{na}$. Results associated with large and small targets are plotted in different colors, in order to distinguish the ID interval in which they lie. However, all IDs were used for the estimation of the index of performance (IP). As expected, $\bar{MT}$ increased for both groups as the ID increased. This increase was significant, according to the regression test and Kruskal–Wallis test (KW), in both groups and conditions (*p* < 0.001). The KW test does not require any previous data requirements, such as normality or homoscedasticity, and can be used for an unbalanced number of subjects among groups.

Table 5 presents the values for the index of performance (IP) and throughput (T). For the latter parameter, it was necessary to previously apply the Weldford correction, in order to adjust the target sizes and distances to experimental data and, with them, the effective index of difficulty ($ID_{e}$) (Equation (9)), which is included in Equation (8) for computing T.

**Figure 10:**
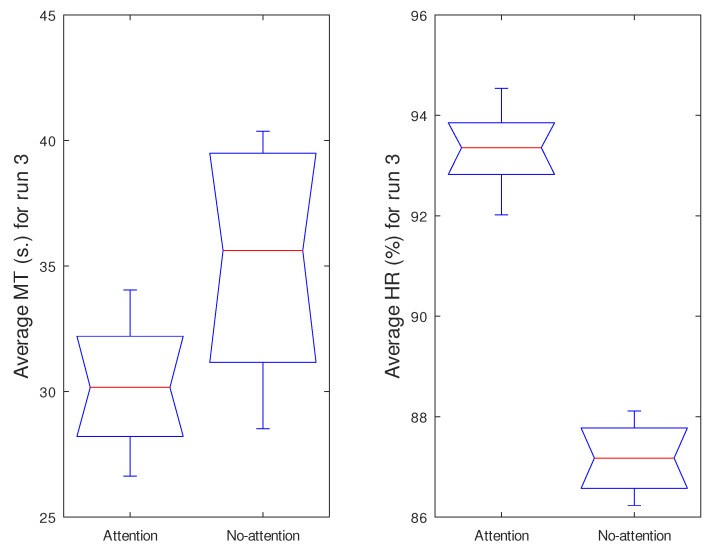
$\bar{MT}$ and $\bar{HR}$ for run 3 in the Attention and No-Attention groups.

One research goal was to determine whether the use of the attention level as a control variable had a significant effect on the cursor movement time and hit rate. Figure 10 shows the boxplots containing the $\bar{MT}$ and $\bar{HR}$ values for all trials and both groups. As can be seen, the Attention group obtained better MT and HR values: lower movement times and higher hit rates. This was also verified statistically, by using the KW, which resulted in p<0.01 and p<0.001 for MT and HR, respectively. This means that the use of attention is a key determinant in obtaining better results in this HCI proposal.

### 4.3. Differences between Groups

To look into the reasons that caused these differences, the target size was included in the following within- and between-group comparisons. Table 6 includes the HR data indexed by ID and condition, as Table 4 does for MT. Both tables contain relevant information for the analysis. Additionally, Table 7 includes the *p*-values obtained after comparing different situations.

As expected, the target size influenced the MT differences within each group in both the fast and averaged conditions (*p* < 0.001). However, when comparing the MT obtained using only big targets between both groups, there were no significant differences, regardless of the condition (fast or averaged). There were only significant differences in MT for small targets (*p* < 0.01) in the averaged condition. Therefore, with small targets, the MT was higher in general; however, when compared along with the attention, it influenced the MT differences between groups only for the averaged condition, not for the fast one.

Regarding HR, there were no significant differences within groups in any condition. We only found differences in the averaged condition between groups for big and small targets (*p* < 0.05 and *p* < 0.01, respectively). Therefore, the target size did not influence HR, regardless of the condition (i.e., fast or averaged). The use of attention as a means of controlling the cursor speed was the key factor, in the averaged condition, that led to the worse results for HR in the $G_{na}$ group.

### 4.4. Metrics Related to the Trajectory

In this section, we detail the analysis of some factors related to the trajectory, such as normalized effective distance (NED), movement variability (MV), movement error (ME), and movement offset (MO), described in [57] and reproduced in Section 3.3.

As expected, the NED was higher than the ideal result—that is, close to 1.2 on average—for both groups, which means that the real path was 20% longer than the optimal one (Table 8). Interestingly, this parameter decreased as ID increased (*p* < 0.001; Table 9), indicating that the participants were less accurate in selecting the initial cursor direction when the target size was large or close. In fact, for big targets, the NED obtained significantly higher values than for small targets, regardless of the group (*p* < 0.001). Here, the attention group obtained slightly better results (*p* < 0.05), which could be mainly explained by the differences obtained between big targets (*p* < 0.05).

**Figure 11:**
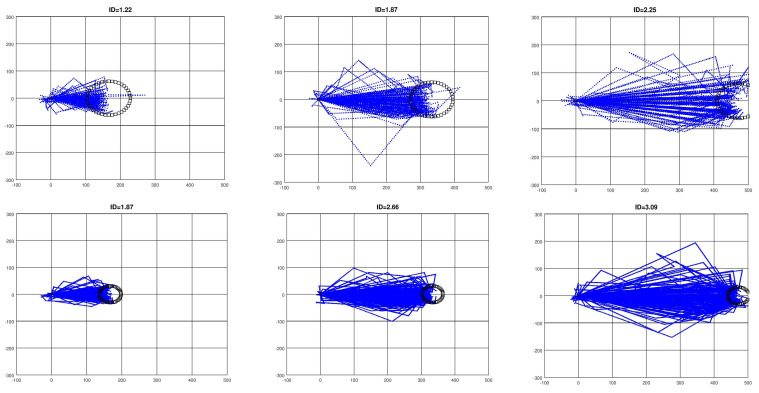
Cursor trajectories for all participants. Low, medium, and high IDs for each target size were chosen. The horizontal axis contains the optimal path between cursor and target, such that the initial cursor position is placed at the origin. As ID increases, the target appears further to the right. The upper row contains trajectories for the big target, while the lower row shows the small targets, which were associated with higher ID values.

Figure 11 shows the cloud of trajectories for all participants, target sizes, and for high, low, and medium index of difficulty (ID) values. To make this possible, as the targets appeared sparsely on the computer during the experiment, the real cursor trajectories are projected over the optimal path, which is the line that joins the cursor’s initial position with the center of the target. In this new representation, such a line is placed horizontally onto the *x*-axis and, consequently, the starting cursor position is moved to the origin of the coordinates. Outliers were not removed from the trajectories, in order to show a complete picture of the experimental results.

As reaching the target involves first selecting the angle at which the cursor leaves the rotation state to initiate its linear movement, it is expected that the real trajectory moves away from the optimal path as the cursor comes closer and closer to the target. As can be seen in Figure 11, these paths drew a kind of cone, with the vertex placed at the cursor’s initial position. This influenced the NED and led to MV and ME having values which indicate that the trajectory diverged from the optimal one by some number of pixels.

Both MV and ME increased as ID did for each target size (*p* < 0.001; Table 9), and the differences were significant when comparing the $G_{a}$ and $G_{na}$ groups. This was, again, due to the differences found between the two groups with the big targets. A possible explanation of this lies in the fact that, once the target size is fixed, the ID increases as the distance does, while the cone aperture angle does not decrease sufficiently, so the real trajectory maintains similar MV and ME values.

MO showed negative values for all groups and IDs, with the exception of one case. On average, the results indicate that there was a negative bias in all trajectories, as can be observed in Figure 11. This means that, in general, there was an anticipation effect when selecting the initial angle. Here, no significant differences between groups and target sizes were found. There was only a clear dependence of MO on ID for the $G_{na}$ group (*p* < 0.01).

### 4.5. Additional Metrics

We also analyzed other parameters related to the cursor movement and its control: the averaged number of linear segments ($\bar{NLin}$), percentage of time in the rotation state ($\bar{PRS}$), and the number of extra actions ($\bar{EA}$). In an ideal situation, only two actions are required to lead the cursor to the target. The first one is selecting the appropriate departure angle, which makes the cursor leave the rotation state and start the linear movement; the second action is selecting the target once the cursor is on it. Therefore, in the best scenario, there are only two states (spin and linear), in a percentage that depends on the initial cursor position and the target placement, while NLin and EA must be 1 and 0, respectively. Table 10 presents the results obtained for both groups.

Similarly to previous sections, Table 11 provides the *p*-values and the significance after comparing the effect of different independent variables on the analyzed metrics. As can be seen, the ID significantly influenced all these variables for both groups (*p* < 0.001), and only for $\bar{PRS}$ were no significant differences found between the $G_{a}$ and $G_{na}$ groups for big, small, and all target sizes.

It can be seen, from the grand averages, that the cursor was in the spinning state almost 50% of the time, and that there was an inverse relationship between PRS and ID, which was statistically significant. The latter was expectable, since as the ID increases, the target is placed further away from the initial cursor position and it takes longer for the cursor to reach it. Interestingly, the use of attention did not have any significant influence on the difference (47.6% and 46.9%, respectively) obtained between the two groups on PRS (*p* > 0.05).

With respect to NLin, Table 10 shows that, in general, it was necessary to use more than one linear segment (and, consequently, rotation states) to reach the target. The ID, target size, and the attention variable had statistically significant influences on this indicator. Therefore, the $G_{na}$ group required more complex trajectories, with more linear segments than the other group (1.79 vs. 1.47); big targets needed less complex trajectories, and, as the ID increased, so did NLin.

EA displayed the same behavior as NLin. In general, subjects had to perform more than one extra action to complete the experiment. For the $G_{na}$ group, the EA was significantly higher (*p* < 0.001), and the use of a large target required fewer extra actions than the small ones (*p* < 0.001) for both groups.

### 4.6. SAM Test and Survey

Participants were asked to complete two types of tests. The first one comprised the SAM test, which involved analyzing their emotions during the experiment, and a set of adjectives or comments that the participants were allowed to add to provide a more complete picture of their feelings. The other survey took place at the end of the experiment. It involved a general assessment related to the participants’ ability to control the cursor’s movement (Q1), the selection of the target (Q2), or the attention level (Q3).

**Figure 12:**
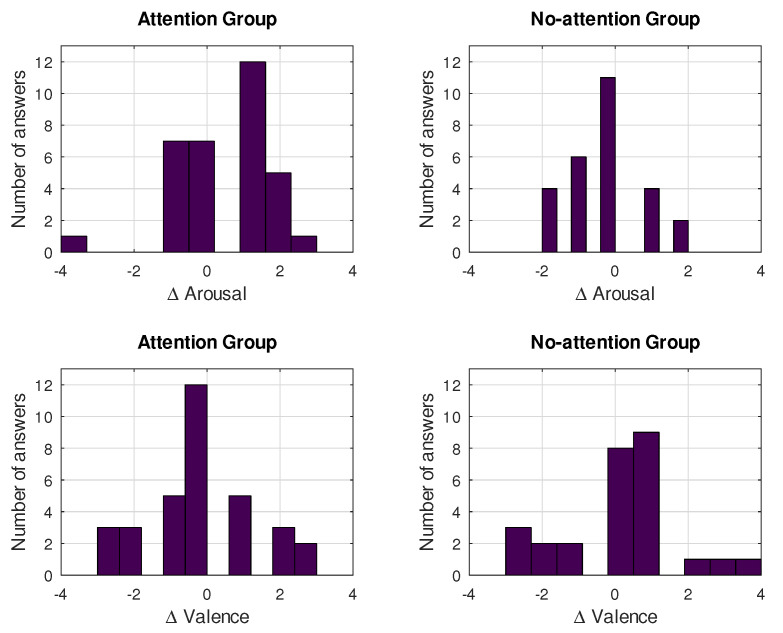
Histograms of differential valence and arousal for Attention and No-Attention groups.

The effect that the use of the proposed method exerted on participants can be quantified by the changes reported in valence and arousal at the end of each run, in relation to those reported at its beginning. This differential (positive or negative) valence and arousal for both groups and all runs is depicted in Figure 12. Most answers suggest differential arousal and valence greater than or equal to zero for both groups; namely, for differential valence, 66.7% and 74.1% of answers in the $G_{a}$ and $G_{na}$ group, respectively, were positive or equal to zero. The $G_{na}$ group had a higher percentage, but this inter-group difference was not statistically significant (*p* = 0.5). Following the same procedure for differential arousal, the respective percentages were 75.8% ($G_{a}$ group) and 63% ($G_{na}$ group); however, it was found that the difference between groups was significant (*p* < 0.05), thus suggesting an effect of the use of attention, leading to an increase in arousal.

**Figure 13:**
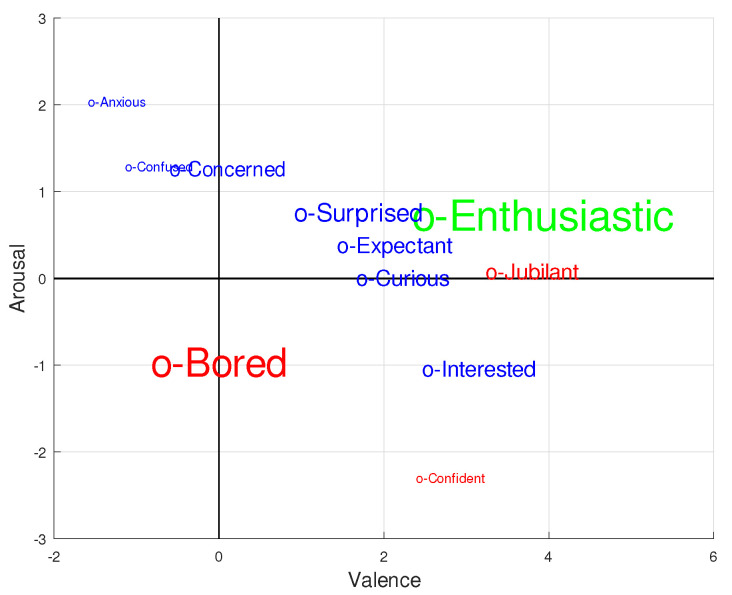
Emotional words obtained from the SAM test results and the coordinates reported in [58], using the bidimensional Russell’s model. Font sizes are proportional to word frequency, and the color shows the group in which the word appeared more frequently: Attention group, blue; No-Attention group, red; same frequency in both groups, green.

Figure 13 shows information collected from the SAM test and translated into emotional words, thanks to [58]—a study that contains a comprehensive list of these emotional words, along with their associated valence and arousal values. By using the absolute arousal and valence scores reported at the end of the three runs for the 20 participants, we found the closest words in the aforementioned list and then constructed a word histogram. The font size of the words appearing in Figure 13 is proportional to their frequency in the histogram. At the same time, with the aim of identifying the group in which a specific word appeared more frequently, three colors were chosen: blue, when a word appeared more times in the $G_{a}$ group; red, when it had a higher frequency in the $G_{na}$ group; and green, when it was equally likely in both groups. The most frequent word was ’enthusiastic’, which appeared in 23.3% of tests and was equally distributed between the two groups. It was followed by ’bored’ (21.7%), which was more frequent in members belonging to the $G_{na}$ group. By far, the largest group of words had frequency close to 8.3%: ‘surprised’, ‘expectant’, ‘curious’, ‘interested’, ‘jubilant’, and ‘concerned’. Excluding ‘jubilant’, which only appeared in the $G_{na}$ group, the remaining words were dominant in the $G_{a}$ group. Finally, with a residual appearance (1.7%), there were words such as ‘anxious’, ‘confident’, and ‘confused’. All of these results suggest that most participants reflected positively on the experience.

Most participants did not report any additional comment (75%); others reported discomfort generated by the ear clip (5%) of the headset, that their eyes felt dry (10%) when using the system for a long time, or about the frustration they felt when the system did not detect their blinks properly (15%), causing a delay in completing the task.

**Figure 14:**
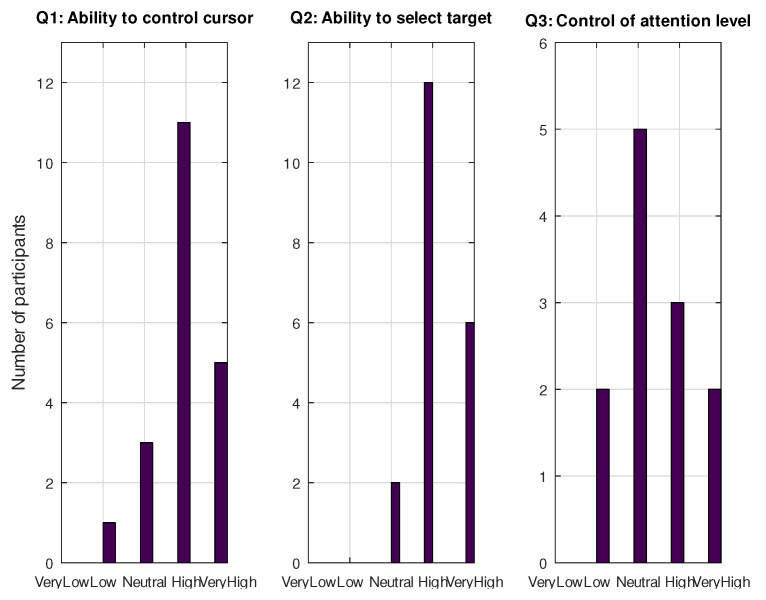
Histograms for collected answers in the final survey.

Figure 14 shows the histograms of the last questionnaire. In general, participants were able to manage the two cursor actions (i.e., movement control and selection). Only one out of the 20 participants (5%) reported that their ability to control the cursor movement was low (Q1), and none of them reported a low or very low capacity in selecting the target (Q2). For those who belonged to the Attention group, only two participants admitted their low capacity for controlling the attention level (16.7%).

## 5. Discussion

To put our proposed approach into context, typical values of HR, MT, IP, and T, for a variety of input devices, were collected. Table 12 shows these values, sorted by IP. At the top of the table, we can find devices such as ETIs, stylus, and mouse, with the highest bit rates, whereas the BCI-based systems occupy the bottom part of the list—where our approach is placed, thus improving upon [19], due to its higher HR and lower MT. There are not many studies that include Fitts’ model of BCI systems for controlling a cursor, which makes it difficult to compare our solution with this technology.

**Figure 15:**
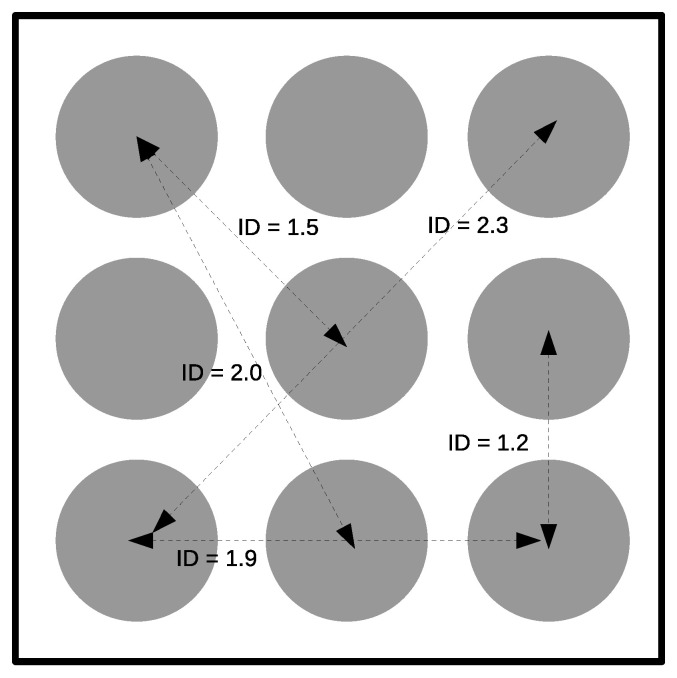
Communication board containing 3 × 3 ideograms symmetrically distributed throughout the application window. With only five different movements, the selection of any ideogram is possible. The ID of each movement is also shown.

To compare the throughput (T) for different BCI systems, the information transfer rate (ITR) was introduced [62,63]. ITR is based on the classic Shannon’s Information Theory. It measures the capacity of a BCI channel in bits/min, which depends on the number of choices, the accuracy of the classifier in detecting them, and the time required to make a classification. In order to extend the comparison with the BCI systems, without adding another experiment, we used the hit rate and movement time obtained previously in the Fitts’ model and a hypothetical communication board, based on a 3 × 3 grid of circle-shaped ideograms (see Figure 15). The ideograms were placed while obeying the distances shown in Table 2, and with a diameter fixed to one-fourth of the screen border.

Table 13 contains the values of $HR_{id}$ and $MT_{id}$ for every ID. Assuming that all ideograms are equally likely, then the $\bar{HR}$ and $\bar{MT}$ averages can be obtained by weighting their respective $HR_{id}$ and $MT_{id}$ by the relative number of all possible trajectories in each ID. Then, the $\bar{HR}$ would act as the probability of selecting one out of *N* ideograms, and $\bar{MT}$ is used as the average time in its selection. Therefore, an estimation of $\hat{ITR}$ can be given by Equation (16).

(14) $$\begin{array}{c} \bar{HR}=\sum_{id}HR_{id}p_{id}, \end{array}$$

(15) $$\begin{array}{c} \bar{MT}=\sum_{id}MT_{id}p_{id}, \end{array}$$

(16) $$\hat{ITR}=\frac{log_{2}N+\bar{HR}\;log_{2}\left(\bar{HR}\right)+\left(1-\bar{HR}\right)log_{2}\left(\frac{1-\bar{HR}}{N-1}\right)}{\bar{MT}}.$$

For a communication panel with 3 × 3 elements, such as that shown in (Figure 15), the ITR is in the range of 2.3–16 bits/min, with 7 bits/min on average. According to [64], the typical ITR for MI and P300 is 3–35 bits/min and 20–25, respectively. The highest score is achieved by SSVEP, which reaches a bit rate of 60–100 bits/min. Therefore, our proposed approach would be placed within the range of MI and P300 BCI systems.

Analysis of the trajectory showed that that of NED was slightly superior (over 20%) to that of the optimal. This optimal, or ideal path, was assumed to have a length of D−R (*D* is the distance between the initial cursor position and the center of the target of radius, *R*). This also requires a perfect departure angle, which allows the cursor to draw a trajectory perpendicular to the target, and the selection must be performed precisely at the target’s border. In order to analyze the behavior of NED for different IDs, a theoretical model was developed. To do so, it was assumed that all possible trajectories that can be followed to successfully reach the target are equally likely. The theoretical model of $NED_{th}$ depends on *D* and *R*, according to Equation (17).

(17) $${\bar{NED}}_{th}=\frac{D-R}{3R}\left[-1+{\left(\frac{D+R}{D-R}\right)}^{1/3}\right].$$

Table 14 compares the results achieved (Table 11) with the values obtained through Equation (17). As can be seen, NED diminishes as ID increases. This is a consequence of the fact that longer distances make the ratio closer to 1. For ID greater than 1.9, $NED_{a}$ and $NED_{na}$ approached $NED_{th}$; for lower values, the experimental result improved upon the theoretical. This means that it was likelier to select trajectories nearer the optimal path for low IDs, considering all the possible trajectories. For high IDs, the set of experimental trajectories matched the theoretical approach reasonably well. This can be explained by the existence of a target ’departure angle’, which is different from the viewing angle that was used in the theoretical model, and which gathers all possible trajectories ending in the target. For low IDs, the viewing angle is greater than the departure angle, which causes $NED_{th}$ to increase. For ID greater than 2.0, the theoretical estimation and experimental data converged reasonably well. This suggests the existence of a minimum departure angle, which seems difficult to reduce. For the No-Attention group ($G_{na}$) and ID greater than 2.7, the theoretical result was less than the experimental; for the other group, this situation occurred for an ID greater than 3.1. As a direct consequence of this fact, it will be more likely, for high ID, that more extra actions (EA) and linear segments are needed, in order to correct the trajectory (Table 10).

In [57], the trajectories of different typical pointing devices, such as mice, trackballs, and so on, were assessed. We replicated their results in Table 15, along with those obtained by our proposed approach. It can be seen that the performance of the cursor’s guidance was similar to that of common pointing devices.

Another issue, concerning the movement time (MT), is that the cursor spun close to 50% of the total time. Several factors make this percentage important. First, the cursor always spins counterclockwise. Therefore, depending on the target position, it is sometimes necessary to wait for almost a whole turn to select the appropriate departure angle. Second, it is not easy to select an appropriate departure angle, especially for high ID, so it is probable that the trajectory has to be corrected again, forcing the need for another spinning state. A simple solution could avoid excessive MT in this situation. This consists of anticipating the selection of the departure angle (or, in other words, selecting a departure angle slightly smaller than the appropriate angle). By doing so, when the cursor’s direction has to be corrected, the target is placed on its left. The counterclockwise rotation can then help the cursor point to the target very quickly. An explanation of why the experimental MO was negative might stem from intuitive learning by the participants, regarding this efficient mode of steering the cursor. Third, the cursor spins discretely in different step angles, $\theta_{step}$, every 0.9 s (the time assumed necessary for the system to detect a double blink). This $\theta_{step}$ was initially set to 13^o^, meaning that the cursor takes 25 s to complete a turn. To reduce this time, a possible solution would be to make $\theta_{step}$ greater—for example, 25—. In spite of the fact that there will be a decrease in rotation time, it is not clear that this will benefit the movement time, as a higher number of trajectory corrections might be needed, especially for small and far targets. To address this question, we conducted a preliminary single-run study, without attention control of speed, and with an increased rotation frequency, approximately twice the original (i.e., it now takes only 12 s to complete a turn). Table 16 shows the new results, along with the previous ones. As can be seen, there was a reduction in $\bar{MT}$ of more than 30%, without a loss in $\bar{HR}$. This suggests that there may be still a margin to improve the proposed approach and increase both the ITR and T. Nevertheless, a study must be conducted with more participants and using statistical tools, in order to extract robust conclusions.

BCI studies usually show the averaged time of selecting elements on the screen. For example, in [12], the authors proposed an SSVEP–BCI system which obtained an MT value of 21.3 s; in [24], the authors developed a hybrid approach combining P300, MI, and SSVEP in two different designs, where the best design achieved an MT of 19 s. Another approach, based on EOG and EEG [32], obtained an averaged time of 19.9 s. In our study, it was possible to obtain an $\bar{MT}$ of 23.2 s using only large targets (or low ID), which was similar to the aforementioned works, and we determined that it is also possible to reduce this time down to 17.5 s by simply doubling the spinning frequency.

It is remarkable that most participants (people without disabilities) reported that they were able to control the cursor’s movements and make a selection properly. Only one of them admitted that they had experienced difficulty, and, in the $G_{a}$ group, two participants declared their low capacity in controlling the cursor speed through the modulation of their attention level. According to the SAM test, 75% of participants reported positive incremental valence at the end of all runs, and two-thirds of participants defined their final state using positive words, such as ‘enthusiastic’, ‘interested’, ‘confident’, and so on. In contrast, the most important negative emotional word was ‘bored’, appearing with a frequency of 21.7%.

The duration of the experiment, which may widely vary among studies, is also an important variable to take into account, as it may have a relative influence on the fatigue, tiredness, or/and boredom of participants. Consequently, it was difficult to compare our results with those of other similar studies. In [32], the authors performed a workload evaluation through the NASA-TLX questionnaire [65], which assesses factors such as mental demand, effort, and frustration level. Ten participants who performed several tasks with a hybrid BCI, including the use of a web browser or writing text, among others, which took them a few minutes to complete, scored under 40% on average, indicating a moderate to high mental demand and effort. Although, on average, the results obtained can be considered acceptable [66], it would have been very interesting to analyze their use for a longer period of time. In spite of the fact that our approach took around 27.6±5.2 min to complete a run, our 20 participants scored the experience positively in a percentage superior to 75%.

A final remark regarding the attention level is that it was a key factor to obtaining better figures. We believe that, aside from modulating the cursor speed, it contributed to better navigation in general, involving a lower number of errors, better departure angle selection in the spinning state, and so on. All of these factors influenced the movement time. Further research must be performed to verify this hypothesis.

## 6. Conclusions and Further Work

This study showed that the use of a low-cost single-electrode EEG headset for controlling a mouse pointer is feasible and reliable. The approach was based on the use of an algorithm that combines two input independent variables: blinks, to control the displacement of the cursor and selections, and attention level, to speed up the cursor movements.

The obtained performance indicators were similar to those of BCI solutions such as MI, while some of the trajectory indicators were in the range of conventional pointing devices, such as trackballs, joysticks, and so on. It was also shown that the movement time can be improved without any negative effect on the selection rate; however, further research must be conducted to support this assertion. The Attention group obtained lower movement times (MT; meaning higher speed/attention values, on average), higher hit rates (HR), better trajectory indicators, and fewer commands (sequence of blinks) needed to complete the task, but they also showed a higher level of arousal. People who participated in the experiment scored it positively, as most words obtained in the SAM test were positive; only one participant of 20 found it difficult to control the cursor.

In the future, we intend to analyze which navigation factors caused the inter-group differences and how they were influenced by the attention level. Additionally, we are in the process of developing our own algorithms to obtain the attention level from neural activity.

## Figures and Tables

**Table 1:** Final survey to assess participants’ perceptions in controlling the cursor’s movements.

1	Score your ability to control cursor movements
2	Score your ability in selecting the target
3	Score your ability to control the attention level ($G_{na}$ excluded)

**Table 2:** Conditions in the experiment conducted to obtain the Fitts’ model. The diameter of the target, W=(2R), and the distance to the initial position of the cursor, *D*, are normalized to the size of the window shown on the computer screen. Two ID’s, marked with an asterisk, were identical for the two target sizes.

*W*	*D*	*ID*
1/4	1/3	1.2
1/4	$\sqrt{2}$/3	1.5
1/4	2/3	1.9 *
1/4	$\sqrt{5}$/3	2.0
1/4	2$\sqrt{2}$/3	2.3 *
1/8	1/3	1.9 *
1/8	$\sqrt{2}$/3	2.3 *
1/8	2/3	2.7
1/8	$\sqrt{5}$/3	2.8
1/8	2$\sqrt{2}$/3	3.1

**Table 3:** $\bar{MT}$ and $\bar{HR}$ for different runs and trials. Averages for each run are also shown.

	Attention						No-Attention				
	Run	$T_{1}$	$T_{2}$	$T_{3}$	$T_{4}$	Ave.	$T_{1}$	$T_{2}$	$T_{3}$	$T_{4}$	Ave.
$\bar{MT}$ (s.)	1	29.5	34	36.3	42.4	35.6	33.2	36.2	41	42.1	38.1
	2	27.1	30.2	34.1	34	31.4	28.5	35.5	42.3	39.5	36.5
	3	26.6	28.7	31.6	34	30.3	28.5	32	39.2	40.4	35
$\bar{HR}$ (%)	1	90.7	91.7	94.8	90.5	91.9	90.6	95.4	95	89.8	92.7
	2	92.5	89.5	87	91.9	90.3	90.3	90.9	82.7	89.9	88.5
	3	93.1	94.5	93.6	92	93.3	88.1	87.7	86.7	86.2	87.2

**Table 4:** $\bar{MT}$ for $G_{a}$ and $G_{na}$ groups (*a* and na subscripts) indexed by ID: ${\bar{MT}}_{fa}$/${\bar{MT}}_{fna}$ (only trial 1) and ${\bar{MT}}_{a}$/${\bar{MT}}_{na}$ (average of all trials). The symbols ○ and *o* represent the big and small targets respectively.

	ID								
	**Target (W)**	**1.2**	**1.5**	**1.9**	**2.0**	**2.3**	**2.7**	**2.8**	**3.1**
${\bar{MT}}_{a}$ (s)	○	17.6	19.4	25.5	28.0	30.7	–	–	–
	o	–	–	24.3	–	29.4	39.0	41.1	47.2
${\bar{MT}}_{na}$ (s)	○	20.2	23.2	29.7	30.2	37.5	–	–	–
	o	–	–	27.0	–	34.0	46.3	49.0	54.5
${\bar{MT}}_{fa}$ (s)	○	15.0	16.9	26.7	26.6	29.1	–	–	–
	o	–	–	19.2	–	27.5	32.9	32.1	40.4
${\bar{MT}}_{fna}$ (s)	○	12.4	18.4	28.5	22.1	32.2	–	–	–
	o	–	–	19.3	–	30.3	34.6	43.0	46.9

**Table 5:** IP and T for different groups and conditions (fast condition is represented by the subscript *f*). Units are in bits per second (bps).

Group	IP	*T*	${\mathrm{IP}}_{f}$	$T_{f}$
Attention	0.061	0.075	0.078	0.085
No-Attention	0.051	0.063	0.055	0.078

**Table 6:** $\bar{HR}$ for $G_{a}$ and $G_{na}$ groups (*a* and na subscripts), for trial 1 (*f* subscript) or all trials (without additional subscript), and indexed by ID. The symbols ○ and *o* represent the big and small targets respectively.

	ID								
	**Target (W)**	**1.2**	**1.5**	**1.9**	**2.0**	**2.3**	**2.7**	**2.8**	**3.1**
${\bar{HR}}_{a}$ (%)	○	94.3	95.3	92.8	90.2	91.2	–	–	–
	o	–	–	97.0	–	92.6	88.8	94.3	96.8
${\bar{HR}}_{fa}$ (%)	○	95.5	95.8	81.1	81.9	93.8	–	–	–
	o	–	–	100.0	–	91.7	100	90.9	95.8
${\bar{HR}}_{na}$ (%)	○	91.9	88.7	80.8	90.6	84.8	–	–	–
	o	–	–	88.3	–	90.3	86.2	84.0	85.6
${\bar{HR}}_{fna}$ (%)	○	100	93.8	76	93.8	75	–	–	–
	o	–	–	90.5	–	85.4	100	83.3	81.3

**Table 7:** *p*-values obtained after applying KW to MT and HR for averaged and fast conditions. The second and third columns explore the existence of significant effects of ID on these parameters in the $G_{a}$ and $G_{na}$ groups. The fourth and fifth columns compare the effect of target size in each group, while the remainder of the columns compare the effect of the use (or not) of attention for big, small, and all target sizes (‘*b*’, ‘*s*’, and ‘all’, respectively).

	${\mathrm{ID}}_{a}$	${\mathrm{ID}}_{\mathrm{na}}$	$B_{a}-S_{a}$	$B_{\mathrm{na}}-S_{\mathrm{na}}$	$B_{a}-B_{\mathrm{na}}$	$S_{a}-S_{\mathrm{na}}$	${\mathrm{All}}_{a}-{\mathrm{All}}_{\mathrm{na}}$
MT	**<0.001**	**<0.001**	**<0.001**	**<0.001**	0.12	**<0.001**	**<0.001**
MT${}_{f}$	**<0.001**	**<0.001**	**<0.001**	**<0.001**	0.16	0.22	0.86
HR	0.44	0.76	0.53	0.85	**<0.05**	**<0.01**	**<0.001**
HR${}_{f}$	0.3	0.34	0.16	0.92	0.63	0.06	0.11

**Table 8:** Results obtained for the trajectory indicators (NED, MV, ME, and MO), indexed by ID, target size, and group. Averages are also shown (on the right). The symbols ○ and *o* represent the big and small targets respectively.

	ID										
	**Target (W)**	**1.2**	**1.5**	**1.9**	**2.0**	**2.3**	**2.7**	**2.8**	**3.1**	**Ave**	
${\bar{NED}}_{a}$	○	1.37	1.22	1.18	1.18	1.14	–	–	–	1.22	1.17
	o	–	–	1.17	–	1.14	1.1	1.09	1.08	1.12	
${\bar{NED}}_{na}$	○	1.44	1.26	1.21	1.19	1.21	–	–	–	1.26	1.2
	o	–	–	1.15	–	1.25	1.11	1.11	1.12	1.15	
${\bar{MV}}_{a}$	○	5.7	7.72	13.3	14.2	13.8	–	–	–	10.9	10.6
	o	–	–	6.12	–	8.26	10.4	13.1	13.5	10.3	
${\bar{MV}}_{na}$	○	7.68	10.5	16.3	16.3	19.7	–	–	–	14.1	13.2
	o	–	–	4.83	–	10.2	13.5	15	18.2	12.3	
${\bar{ME}}_{a}$	○	3.34	5.62	10.1	13	13.3	–	–	–	9.06	10.7
	o	–	–	5.25	–	10.2	12.3	15.5	18.8	12.4	
${\bar{ME}}_{na}$	○	5.26	7.66	16.2	17.3	24.2	–	–	–	14.1	14.2
	o	–	–	4.34	–	11	18.7	17.2	20.4	14.3	
${\bar{MO}}_{a}$	○	−2.96	−3.67	−4.37	−7.41	−6.85	–	–	–	−5.05	−4.93
	o	–	–	−2.37	–	−6.1	−5	−3.66	−6.94	−4.81	
${\bar{MO}}_{na}$	○	−3.47	−4.25	−1.98	−5.39	−11.8	–	–	–	−5.38	−3.3
	o	–	–	−2.44	–	−1.61	9.66	−3.02	−8.73	−1.23	

**Table 9:** *p*-values obtained after analyzing the statistical influence of different conditions on NED, MV, ME, and MO. The second and third columns compare the existence of any significant effect of ID on these parameters in each group. The fourth and fifth columns compare the effect of target size within each group, while the remaining columns compare the effect of the use (or not) of attention for big (B), small (S), and all (All) target sizes.

	${\mathrm{ID}}_{a}$	${\mathrm{ID}}_{\mathrm{na}}$	$B_{a}-S_{a}$	$B_{\mathrm{na}}-S_{\mathrm{na}}$	$B_{a}-B_{\mathrm{na}}$	$S_{a}-S_{\mathrm{na}}$	${\mathrm{All}}_{a}-{\mathrm{All}}_{\mathrm{na}}$
NED	**<0.001**	**<0.001**	**<0.001**	**<0.001**	**<0.05**	0.1	**<0.05**
MV	**<0.001**	**<0.001**	0.79	0.3	**<0.01**	0.087	**<0.01**
ME	**<0.001**	**<0.001**	**<0.01**	0.61	**<0.001**	0.18	**<0.001**
MO	0.15	**<0.01**	0.89	0.075	0.97	0.05	0.16

**Table 10:** Additional metrics for cursor movement and control: percentage of time that cursor is in the rotation state ($\bar{PRS}$), averaged number of linear segments contained in the trajectory ($\bar{NLin}$), and averaged number of extra actions ($\bar{EA}$). Grand averages are also shown on the right.

	ID										
	**Target (W)**	**1.2**	**1.5**	**1.9**	**2.0**	**2.3**	**2.7**	**2.8**	**3.1**	**Ave**	
${\bar{PRS}}_{a}$	○	66.1	56.1	55.1	50	43.5	–	–	–	54.1	47.6
	o	–	–	51.9	–	44.2	39.3	37.2	32.4	41	
${\bar{PRS}}_{na}$	○	64	57.5	49.8	47	44.7	–	–	–	52.6	46.9
	o	–	–	49.6	–	42.6	40.8	39.9	33.7	41.3	
${\bar{NLin}}_{a}$	○	1	1	1.37	1.57	1.42	–	–	–	1.27	1.47
	o	–	–	1.45	–	1.58	1.8	1.84	1.64	1.66	
${\bar{NLin}}_{na}$	○	1.34	1.29	1.7	1.66	2.1	–	–	–	1.62	1.79
	o	–	–	1.44	–	2.13	1.93	2.15	2.19	1.97	
${\bar{EA}}_{a}$	○	0	0.455	0.783	1.26	0.867	–	–	–	0.673	1.05
	o	–	–	0.894	–	1.22	1.69	1.98	1.33	1.42	
${\bar{EA}}_{na}$	○	0.793	0.643	1.48	1.41	2.28	–	–	–	1.32	1.75
	o	–	–	1	–	2.4	1.89	2.5	3.07	2.17	

**Table 11:** *p*-values obtained after analyzing the statistical influence of different conditions on PRS, NLin, and EA. The fourth and fifth columns compare the effect of target size in each group, while the remaining columns compare the effect of the use (or not) of attention for big (B), small (S), and all (All) target sizes.

	${\mathrm{ID}}_{a}$	${\mathrm{ID}}_{\mathrm{na}}$	$B_{a}-S_{a}$	$B_{\mathrm{na}}-S_{\mathrm{na}}$	$B_{a}-B_{\mathrm{na}}$	$S_{a}-S_{\mathrm{na}}$	${\mathrm{All}}_{a}-{\mathrm{All}}_{\mathrm{na}}$
PRS	**<0.001**	**<0.001**	**<0.001**	**<0.001**	0.56	0.81	0.81
NLin	**<0.001**	**<0.001**	**<0.001**	**<0.001**	**<0.001**	**<0.01**	**<0.001**
EA	**<0.001**	**<0.001**	**<0.001**	**<0.001**	**<0.01**	**<0.01**	**<0.001**

**Table 12:** Comparison among different studies. The device column lists the type of cursor control and the selection method in parentheses. Devices are sorted according to IP or T, so those with higher scores are placed at the top of the table. ${}^{1}$ T was estimated using the data shown in the manuscript and verified by dividing the average ID into MT.

Device	ID Range (bits)	HR (%)	MT (ms)	IP (bps)	T (bps)	Ref.
ET (dwell)	[1.28, 3.06]	57.1	450	13.8	–	[59]
ET (touch)	[1.28, 3.06]	88.3	570	10.9	–	[59]
Stylus	[1, 6.02]	96	665	4.9	–	[60]
Mouse	[1, 6.02]	96.5	674	4.5	–	[60]
Trackball	[1, 6.02]	96.1	1,101	3.3	–	[60]
ET (AL)	[2.4, 4.6]	86.7	2,260	2.27	–	[29,61]
Ankle	[2.2, 3.8]	–	600	1.49	–	[1]
Head (touch)	[1.58, 2.32]	91.6	1,534	–	1.42	[3]
BCI-MI (clench)	[0.58, 3.79]	>90	1780–11,210	0.63	–	[20]
Head (smile)	[1.81, 3.22]	88.4	4,400	–	0.60	[2]
Head (smile)	[1.81, 3.22]	88.4	4,400	–	0.60	[2]
ET-MI	[1, 3.32]	85.1	3,347	–	0.438	[27]
**Ours (att, fast)**	**[1.2, 3.1]**	**93.1**	**26,600**	**0.078**	**0.085**	
**Ours (att)**	**[1.2, 3.1]**	**93.3**	**30,300**	**0.061**	**0.075**	
**Ours (no-att, fast)**	**[1.2, 3.1]**	**88.1**	**28,500**	**0.055**	**0.078**	
**Ours (no-att)**	**[1.2, 3.1]**	**87.2**	**35,000**	**0.051**	**0.063**	
BCI-MI (no click)	[0.58, 3.62]	56.8	40,800	–	0.04 ${}^{1}$	[19]

**Table 13:** HR and MT for big targets and Attention group, along with the probability, $p_{id}$, of performing a movement associated to accessing a target with a specific ID.

*ID*	*HR_id_*	*MT_id_*(*s*.)	*p_id_*
1.2	0.943	17.6	24/72
1.5	0.953	19.4	16/72
1.9	0.928	25.5	12/72
2.0	0.902	28.0	16/72
2.3	0.912	30.7	4/72

**Table 14:** Theoretical and experimental NED for the Attention and No-Attention groups. The symbols ○ and *o* represent the big and small targets respectively.

	ID								
	**Target (W)**	**1.2**	**1.5**	**1.9**	**2.0**	**2.3**	**2.7**	**2.8**	**3.1**
${\bar{NED}}_{a}$	○	1.37	1.22	1.18	1.18	1.14	–	–	–
	o	–	–	1.17	–	1.14	1.1	1.09	1.08
${\bar{NED}}_{na}$	○	1.44	1.26	1.21	1.19	1.21	–	–	–
	o	–	–	1.15	–	1.25	1.11	1.11	1.12
${\bar{NED}}_{th}$	○	1.95	1.44	1.26	1.22	1.16	–	–	–
	o	–	–	1.26	–	1.16	1.11	1.10	1.07

**Table 15:** Some trajectory indicators for common pointing devices, obtained from [57], along with our experimental results for the attention group.

Variable	Mouse	Trackball	Joystick	Touchpad	Ours (Attention)
$\bar{MV}$	10.5	15.9	17.6	11.7	**10.6**
$\bar{ME}$	11.6	16.5	18.7	13.2	**10.7**
$\bar{MO}$	2.5	3.4	5.1	3.9	−4.93

**Table 16:** Comparison between the main performance indicators, HR and MT, with a higher rotation frequency of the cursor. The symbols ○ and *o* represent the big and small targets respectively.

		ID								
	**Target (W)**	**1.2**	**1.5**	**1.9**	**2.0**	**2.3**	**2.7**	**2.8**	**3.1**	**Ave.**
${\bar{MT}}_{na}$ (s)	○	20.2	23.2	29.7	30.2	37.5	–	–	–	28.2
	o	–	–	27.0	–	34.0	46.3	49.0	54.5	42.2
${\bar{MT}}_{hrs}$ (s)	○	12.2	11.6	21.7	19.3	22.6	–	–	–	17.5
	o	–	–	19.2	–	26.8	34.9	24	42.8	29.5
${\bar{HR}}_{na}$ (%)	○	91.9	88.7	80.8	90.6	84.8	–	–	–	87.4
	o	–	–	88.3	–	90.3	86.2	84.0	85.6	86.9
${\bar{HR}}_{hrs}$ (%)	○	87.5	87.5	87.5	100	87.5	–	–	–	90
	o	–	–	87.5	–	83.3	70.8	100	100	88.3

## Abbreviations

The following abbreviations are used in this manuscript:

ALS	amyotrophic lateral sclerosis
BCI	brain–computer interface
c-VEP	code-modulated visual evoked potential
EA	number of extra actions
EEG	electro-encephalography
EOG	electro-oculography
ETI	eye-tracker interface
FSM	finite state machine
HCI	human–computer interaction
HR	hit rate
ID	index of difficulty
IP	index of performance
IR	infrared
ITR	information transfer rate
KW	Kruskal–Wallis test
LIS	locked-in syndrome
LSL	labstreaming layer
ME	movement error
MI	motor imagery
MO	movement offset
MT	movement time
MV	movement variability
NED	normalized effective distance
NM	NeuroSky Mindwave
SAM	self-assessment manikin
SSVEP	steady state visual potential
T	throughput
